# Role of CYP17 rs743572 Polymorphism in Benign Prostatic Hyperplasia: A Multivariate Integrated Analysis

**DOI:** 10.3389/fphys.2019.00774

**Published:** 2019-06-21

**Authors:** Hong Weng, Cheng Fang, Pei-Liang Geng, Ying-Hui Jin, Xian-Tao Zeng, Xing-Huan Wang

**Affiliations:** ^1^Department of Urology, Zhongnan Hospital of Wuhan University, Wuhan, China; ^2^Center for Evidence-Based and Translational Medicine, Zhongnan Hospital of Wuhan University, Wuhan, China; ^3^Center for Evidence-Based and Translational Medicine, Wuhan University, Wuhan, China; ^4^Department of Evidence-Based Medicine and Clinical Epidemiology, The Second Clinical College of Wuhan University, Wuhan, China

**Keywords:** CYP17, polymorphism, meta-analysis, risk factor, benign prostatic hyperplasia

## Abstract

**Objective:** Many published studies have investigated the association between CYP17 rs743572 polymorphism and benign prostatic hyperplasia (BPH) susceptibility but have yielded inconsistent results. Hence, we performed this meta-analysis using the multivariate statistic method to address a more precise association.

**Methods:** Case-control or cohort studies with adequate genotype distribution or minor allele frequency (MAF) were identified by searching the PubMed, Embase, and Web of Science databases up to December, 2018. Odds ratios (ORs) and 95% confidence intervals (CIs) were calculated to estimate the association between CYP17 rs743572 polymorphism and BPH susceptibility.

**Results:** Pooled MAFs of 13 studies were 37% in Caucasians and 56% in Orientals, respectively. Pooled results of 8 studies suggested that CYP17 rs743572 was not associated with the BPH susceptibility in the overall population (OR = 0.98, 95% CI: 0.80–1.20 for A2 vs. A1; OR = 0.99, 95% CI: 0.79–1.25 for A1/A2 vs. A1/A1; OR = 0.97, 95% CI: 0.62–1.53 for A2/A2 vs. A1/A1). Sensitivity analysis showed the results were robust. Subgroup analysis based on ethnicity suggested that, in Orientals, A2 allele carriers had a 28% lower risk of developing BPH compared with A1 allele carriers, and the risk of BPH is 47% lower in A2/A2 genotype carriers compared with A1/A1 genotype carriers. No significant association was observed in Caucasians.

**Conclusion:** In conclusion, our study indicates a negative association between CYP17 and BPH in Orientals. However, due to limited sample size, the conclusion should be interpreted with caution.

## Introduction

Benign prostatic hyperplasia (BPH) is one of the most common benign neoplasms and the most important etiology of lower urinary tract symptoms, causing symptoms in 75% of elderly men (Platz et al., 2002; Priest et al., 2012; Santos Dias, 2012; Vuichoud and Loughlin, 2015; Egan, 2016; Calogero et al., 2018). BPH has been a main concern for higher years lived with disability rates in adult males older than 55 years (GBD 2016 Disease Injury Incidence Prevalence Collaborators, 2017). The disease is characterized by benign enlargement of the prostate gland, resulting in obstruction of the urethra. BPH alone is rarely a fatal condition but can decrease the quality of life. More and more researchers have indicated that BPH may be a new metabolic disease of aging males (Corona et al., 2014; De Nunzio et al., 2014; Vignozzi et al., 2014; Gacci et al., 2015; Li et al., 2018; Shih et al., 2018; Zeng et al., 2018a,b,c). In addition, BPH is a multifactorial and complex disease and its etiology remains unclear. Hence, the need to identify the etiology or risk factors of BPH is urgent and our team has proposed a study protocol to identify the risk factors for BPH (Zeng et al., 2018a).

More researchers have suggested that genetic polymorphisms may play a vital role in the development of BPH (Na et al., 2017; Peng et al., 2017; Su et al., 2017; Zeng et al., 2017). Steroidal hormones (especially androgen and estrogen) play vital roles in the physiological growth and development of the prostate gland. Therefore, any factors influencing the steroidal hormonal levels may impact the BPH susceptibility (Konwar et al., 2008). Moreover, these hormones are metabolized by Cytochrome P450 (CYP) enzymes (e.g., CYP17). Hence, functional polymorphisms in these genes may elevate or decrease the susceptibility of BPH.

The CYP17 gene, which is located on chromosome 10, codes for the Cytochrome P450c17α enzyme (Picado-Leonard and Miller, 1987). In the 5′-untranslated promoter region of the CYP17 gene (the rs743572 polymorphism, http://www.ncbi.nlm.nih.gov/snp/?term=rs743572), a T (A1 allele) to C (A2 allele) substitution has been assumed to eliminate CYP17 gene expression, causing higher levels of androgens. Numerous molecular epidemiological studies have addressed the association between CYP17 rs743572 polymorphism and BPH susceptibility. However, the available evidence is controversial. Moreover, it lacks meta-analysis to confirm the natural association. Therefore, we performed the present meta-analysis based on the multivariate method to evaluate the possible role of CYP17 rs743572 polymorphism in BPH.

## Materials and Methods

### Eligible Criteria

We performed the present meta-analysis according to Preferred Reporting Items for Systematic Reviews and Meta-analyses (PRISMA) statement (Moher et al., 2009). For minor allele frequency (MAF), any human studies (participants should be male and non-BPH) that addressed the prevalence of CYP17 rs743572 polymorphism at codon−34 (A1/A2) and reported on homogeneous ethnicity were included, regardless of other factors such as sample size and type of report. For assessing genetic association, studies were included if they met the following eligible criteria: (1) CYP17 rs743572 polymorphism at codon−34 (A1/A2) were determined (intervention/exposure) and the patients were male (population); (2) the outcome was incident or prevalent BPH (outcome); (3) the study compared BPH vs. control groups, and control groups were non-BPH population (comparator); (4) designed as a case-control or cohort study (study design); (5) the gene distribution or allele frequency were provided.

### Search Strategy

A comprehensive electronic search in PubMed, Embase, and Web of Science was conducted up to December, 2018. For MAF, the search strategy was performed as follows: (“steroid 17 alpha-monooxygenase” OR 17-hydroxylase OR “Cytochrome P450c17” OR “Cytochrome P450 17” OR CYP17) AND (male OR men) AND (polymorphism OR mutation OR genetic OR variant OR haplotype OR “genetic, polymorphism”). For association between gene polymorphism and BPH, the search strategy was performed as follows: (“benign prostate hypertrophy” OR BPH OR “prostatic hyperplasia” OR “prostatic hypertrophy” OR “benign prostatic enlargement” OR “prostatic enlargement”) AND (“steroid 17 alpha-monooxygenase” OR 17-hydroxylase OR “Cytochrome P450c17” OR “Cytochrome P450 17” OR CYP17) AND (polymorphism OR mutation OR genetic OR variant OR haplotype OR “genetic, polymorphism”). Moreover, references in the recent reviews and included articles were identified for any further possibly related studies. The study subjects were human, and no language restriction was applied.

### Data Extraction

Data were extracted independently by two reviewers who used a pre-specified data extraction form. Any discrepancy was resolved by discussion. The following information was extracted: first author, year of publication, country, ethnicity, sample size, and frequency of genotype distribution. For association between gene polymorphism and BPH, additional data were extracted as follows: source of control, genotyping method, and information for quality assessment.

### Methodological Quality Assessment

The quality assessment of included studies was also independently conducted by two authors, using the modified quality assessment scale proposed in previous systematic reviews and meta-analyses of molecular epidemiology studies (Thakkinstian et al., 2005; Zeng et al., 2015a). The quality score ranges from 0 to 15 points (Table 1).

**Table 1:** Scale for quality assessment of molecular association studies of benign prostatic hyperplasia.

**Criteria**	**Score**
**REPRESENTATIVENESS OF CASES**	
Consecutive/randomly selected form case population with clearly defined sampling frame	2
Consecutive/randomly selected form case population without clearly defined sampling frame or with extensive inclusion/exclusion criteria	1
No method of selection described	0
**SOURCE OF CONTROLS**	
Healthy- or population-based	2
Hospital-based or mixed	1
Not described	0
**ASCERTAINMENT OF BENIGN PROSTATIC HYPERPLASIA**	
Clearly described objective criteria for diagnosis of benign prostatic hyperplasia	2
Diagnosis of benign prostatic hyperplasia by patient self-report or by patient history	1
Not described	0
**ASCERTAINMENT OF CONTROLS**	
Controls were tested to screen out benign prostatic hyperplasia, i.e., measured digital rectal examination, transrectal ultrasound, or PSA level	2
Controls were subjects who did not report benign prostatic hyperplasia; no objective testing	1
Not described	0
**HARDY-WEINBERG EQUILIBRIUM IN CONTROLS**	
Hardy-Weinberg equilibrium	2
Hardy-Weinberg disequilibrium	1
No checking for Hardy-Weinberg equilibrium	0
**GENOTYPING EXAMINATION**	
Genotyping done under “blinded” condition	1
Unblinded or not mentioned	0
**ASSOCIATION ASSESSMENT**	
Assess association between genotypes and benign prostatic hyperplasia with appropriate statistics and adjustment for confounders	2
Assess association between genotypes and benign prostatic hyperplasia with appropriate statistics and without adjustment for confounders	1
Inappropriate statistics used	0
**RESPONSE RATE**	
Response rates for both groups are the same, i.e., to within 5%	2
Response rates are different, between 5 and 10%	1
Response rates are more than 10% different, or no mention of response rates	0

### Statistical Analysis

We used three approaches to estimate the gene association for CYP17 rs743572 polymorphism in BPH as previous meta-analysis (Thakkinstian et al., 2012). First, the per-allele approach was used. The A1 and A2 are major and minor alleles for CYP17 gene polymorphism at codon−34, respectively. The A2 minor allele prevalence of CYP17 gene polymorphism in various ethnic groups (Caucasian and Oriental) was estimated by a random effect model if heterogeneity was detected. Odds ratio (OR) for A2 alleles vs. A1 alleles with corresponding 95% confidence interval (CI) were estimated. Second, the per-genotype approach was applied. A1/A1, A1/A2, and A2/A2 are common homozygous, heterozygous, and minor homozygous genotypes for CYP17 gene polymorphism, respectively. Two odds ratios (OR_1_ for A1/A2 vs. A1/A1 and OR_2_ for A2/A2 vs. A1/A1) were estimated by multivariate meta-analysis (multinomial logistic regression), which is a more advanced method for estimating gene-disease associations, supposed by Bagos (2008). At last, the genetic model of inheritance was inferred and measured by a model-free approach with the parameter lambda (λ = log OR_1_/ log OR_2_), which ranges from 0 to 1 (Minelli et al., 2005; Bagos, 2008). If λ equals to 0, a recessive genetic model is suggested; if λ equals to 0.5, a co-dominant genetic model is suggested; if λ equals to 1, a dominant genetic model is suggested; and values >1 or <0 would suggest positive or negative heterosis.

Hardy-Weinberg equilibrium (HWE) was detected in the control group of each study by using appropriate goodness-of-fit χ^2^ test. The between-study heterogeneity was assessed by a *Q* test. *I*^2^ was applied to quantify the degree of heterogeneity (Higgins et al., 2003). The metrics of *I*^2^ > 40% and *P* < 0.1 (for *Q* test) at the same time indicated the existence of heterogeneity and the meta-analysis was performed using random-effect model. Subgroup analyses were performed according to ethnicity, genotyping method, and control source. Sensitivity analyses was conducted by excluding studies not in HWE and removing studies with quality scores <8 points. Publication bias was assessed using Begg's funnel plot and Egger's test (Egger et al., 1997). Analyses were performed using the Stata 12.0 software, and power analyses were conducted using G^*^Power statistical software. A two-sided *P*-value ≤ 0.05 was considered statistically significant except for heterogeneity tests.

## Results

### Study Characteristics

**Figure 1:**
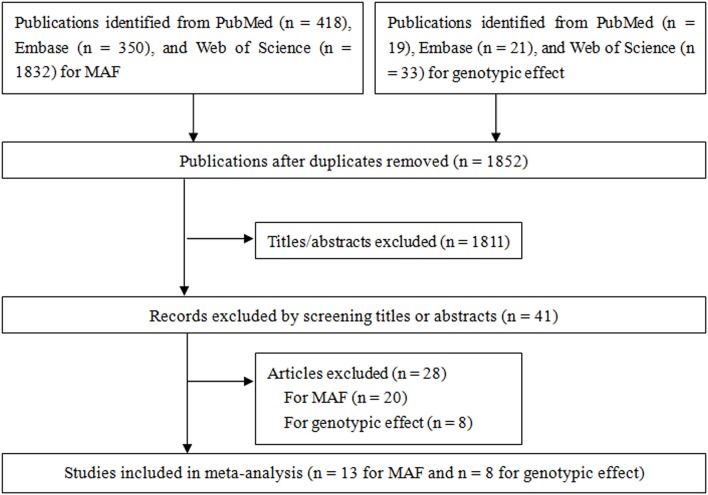
Flow chart from identification of eligible studies to final inclusion.

Figure 1 shows the results of literature search and study. For pooling MAF, a total of 418, 350, and 1,832 studies were identified from PubMed, Embase, and Web of Science, respectively, of which 13 case-control studies (Habuchi et al., 2000; Allen et al., 2001; Haiman et al., 2001; Zmuda et al., 2001; Madigan et al., 2003; Tigli et al., 2003; Gunes et al., 2007; Onen et al., 2007; Sobti et al., 2008; Souiden et al., 2011; Antognelli et al., 2013; El Ezzi et al., 2014; Kumar et al., 2014) reporting the MAF in non-BPH populations in two ethnicities (Caucasian and Oriental) were included for pooling minor allele prevalence. For gene effect, a total of 19, 21, and 33 studies were identified from PubMed, Embase, and Web of Science, respectively, of which eight case-control studies (Habuchi et al., 2000; Madigan et al., 2003; Tigli et al., 2003; Gunes et al., 2007; Sobti et al., 2008; Antognelli et al., 2013; El Ezzi et al., 2014; Kumar et al., 2014) were included in the present meta-analysis for pooling gene effect between CYP17 rs743572 polymorphism and BPH. Of these eight studies, two (Habuchi et al., 2000; Madigan et al., 2003) of them were conducted on Oriental populations and six (Tigli et al., 2003; Gunes et al., 2007; Sobti et al., 2008; Antognelli et al., 2013; El Ezzi et al., 2014; Kumar et al., 2014) on Caucasian populations. The quality score of included studies ranged from 1 to 13 points, and the mean score was 8.6 points. The statistical power of included studies ranged from 22.8 to 92.9%, and the mean power was 43.7%. The estimation of MAF is shown in Table 2 and characteristics of included studies for gene effect are shown in Table 3.

**Table 2:** Estimation of the pooled prevalence of the A2 allele.

**References**	***P* for HWE**	**Total no**.	**A2 allele frequency (no.)**	**% with A2 allele**
**CAUCASIAN**				
Allen et al., 2001	0.33	1,242	439	35
Haiman et al., 2001	0.12	1,564	604	39
Zmuda et al., 2001	0.66	666	263	39
Tigli et al., 2003	0.38	146	52	36
Gunes et al., 2007	0.71	204	58	28
Onen et al., 2007	0.91	210	91	43
Sobti et al., 2008	0.007^a^	340	106	31
Souiden et al., 2011	0.96	250	84	34
Antognelli et al., 2013	0.001^a^	1,160	510	44
El Ezzi et al., 2014	0.005^a^	158	76	48
Kumar et al., 2014	0.07	200	86	43
**ORIENTAL**				
Habuchi et al., 2000	0.55	262	134	51
Madigan et al., 2003	0.22	548	333	61

a Not included in pooling minor allele prevalence.

HWE, Hardy-Weinberg equilibrium.

**Table 3:** The characteristics of studies included in pooling gene effects.

**References**	**Country (ethnicity)**	**Genotyping method**	**Control source**	**Case/Control**	**Cases**			**Controls**			**Power (%)^a^**	**Quality score**
					**A1/A1**	**A1/A2**	**A2/A2**	**A1/A1**	**A1/A2**	**A2/A2**		
Habuchi et al., 2000	Japan (Oriental)	PCR	HB	202/131	74	95	33	33	62	36	44.8	11
Madigan et al., 2003	China (Oriental)	PCR-RFLP	PB	182/274	39	86	57	47	121	106	57.2	12
Tigli et al., 2003	Turkey (Caucasian)	PCR	NR	80/73	34	34	12	32	30	11	23.5	1
Gunes et al., 2007	Turkey (Caucasian)	PCR-RFLP	HB	136/102	48	62	26	53	40	9	33.9	11
Sobti et al., 2008	India (Caucasian)	PCR	HB	170/170	76	86	8	73	88	9	45.5	5
Antognelli et al., 2013	Italy (Caucasian)	PCR-RFLP	HB	588/580	195	262	131	202	246	132	92.9	13
El Ezzi et al., 2014	Lebanon (Caucasian)	PCR-RFLP	Mixed	68/79	21	35	12	15	52	12	22.8	8
Kumar et al., 2014	India (Caucasian)	PCR-RFLP	NR	100/100	26	50	24	28	58	14	29.3	8

a Assuming an odds ratio of 1.5 (small effect size) at α = 0.05 level of significance.

PCR, polymerase chain reaction; PCR-RFL, polymerase chain reaction-restriction fragment length polymorphism; HB, hospital-based study; PB, population-based study; NR, not reported.

### Minor Allele Prevalence

To estimate the pooled frequency (A2), we used data only from a non-BPH population. Three studies (Sobti et al., 2008; Antognelli et al., 2013; El Ezzi et al., 2014) not in HWE were excluded from the pooled analysis for prevalence, leaving eight studies of Caucasians and two studies of Orientals to be pooled. There was moderate to high between-study heterogeneity (*I*^2^ = 64.9%, *P* < 0.001) among the eight Caucasian studies and the pooled MAF using a random-effect model was 37% (95% CI: 34–40%). There was heterogeneity (*I*^2^ = 85.0%, *P* = 0.01) among Oriental studies and the pooled MAF was 56% (95% CI: 47–66%).

### CYP17 rs743572 Polymorphism and BPH Susceptibility

Per-allele approach (A2 vs. A1) showed that there was no significant difference between A2 allele carriers and A1 allele carriers in the total population (OR = 0.98, 95% CI: 0.80–1.20), as well as some evidence of heterogeneity (*I*^2^ = 68.6%, *P* = 0.002). Sensitivity analyses were performed by excluding studies not in HWE and removing low quality studies (<8 points); the results were similar in showing no significant genetic effect and the heterogeneity did not decrease. Subgroup analysis according to ethnicity showed that A2 allele carriers had a ~28% (OR = 0.72, 95% CI: 0.58–0.89) lower risk of developing BPH than individuals with A1 allele in Orientals, with no evidence of heterogeneity (*I*^2^ = 7.0%, *P* = 0.300); and no significant difference was found in Caucasians. Subgroup analyses according to genotyping method and control source are presented in Table 4.

**Table 4:** Determination of the genetic effects of CYP17 gene polymorphism on benign prostatic hyperplasia.

**Genotype**	**Number of studies**	**Sample size (case/control)**	**Test of heterogeneity**		**Test of association**	
			***I*^**2**^ (%)**	***P-*value**	**OR**	**95% CI**
**A2 vs. A1^a^**	8	3,052/3,018	68.6	0.002	0.98	0.80–1.20
HWE (yes)	5	1,400/1,360	81.2	<0.001	1.02	0.71–1.47
Quality score (≥8)	6	2,552/2,532	77.5	<0.001	0.98	0.76–1.28
**ETHNICITY**						
Caucasians	6	2,284/2,208	49.7	0.0077	1.11	0.91–1.35
Orientals	2	768/810	7.0	0.300	0.72	0.58–0.89
**GENOTYPING METHOD**						
PCR	3	904/748	52.9	0.120	0.83	0.61–1.13
PCR-RFLP	5	2,148/2,270	71.5	0.007	1.08	0.80–1.39
**CONTROL SOURCE**						
PB	1	364/548	–	–	0.79	0.60–1.03
HB	4	2,192/1,966	82.8	0.001	1.01	0.72–1.43
**A2/A2 vs. A1/A1^b^**	8	816/812	93.9	<0.001	0.97	0.62–1.53
HWE (yes)	5	373/369	84.6	<0.001	1.07	0.51–2.26
Quality score (≥8)	6	686/687	95.2	<0.001	0.99	0.54–1.80
Caucasians	6	613/590	93.8	<0.001	1.27	0.81–1.99
Orientals	2	203/222	17.2	0.272	0.53	0.34–0.84
**GENOTYPING METHOD**						
PCR	3	237/194	0	0.480	0.45	0.28–0.71
PCR-RFLP	5	579/618	95.1	<0.001	1.62	0.39–6.64
**CONTROL SOURCE**						
PB	1	96/153	–	–	0.65	0.38–1.10
HB	4	591/547	92.6	<0.001	1.68	0.27–10.44
**A1/A2 vs. A1/A1^b^**	8	1,223/1,880	29.9	0.190	0.99	0.79–1.25
HWE (yes)	5	548/504	33.9	0.196	1.01	0.70–1.44
Quality score (≥8)	6	993/957	49.4	0.079	0.99	0.73–1.35
Caucasians	6	929/917	30.9	0.203	1.08	0.82–1.42
Orientals	2	294/263	0.0	0.542	0.76	0.52–1.11
**GENOTYPING METHOD**						
PCR	3	399/318	0.0	0.528	0.86	0.64–1.17
PCR-RFLP	5	824/862	47.4	0.107	1.05	0.86–1.28
**CONTROL SOURCE**						
PB	1	125/168	–	–	0.86	0.52–1.41
HB	4	898/797	49.6	0.114	1.05	0.87–1.28

a The per-allele model (A2 vs. A1) was estimated using bivariate meta-analysis.

b The A2/A2 vs. A1/A1 and A1/A2 vs. A1/A1 genetic models were estimated busing multivariate meta-analysis.

HWE, Hardy-Weinberg equilibrium; OR, odds ratio; CI, confidence interval; PCR, polymerase chain reaction; PCR-RFLP, PCR-Restriction Fragment Length Polymorphism; PB, population-based; HB, hospital-based.

**Figure 2:**
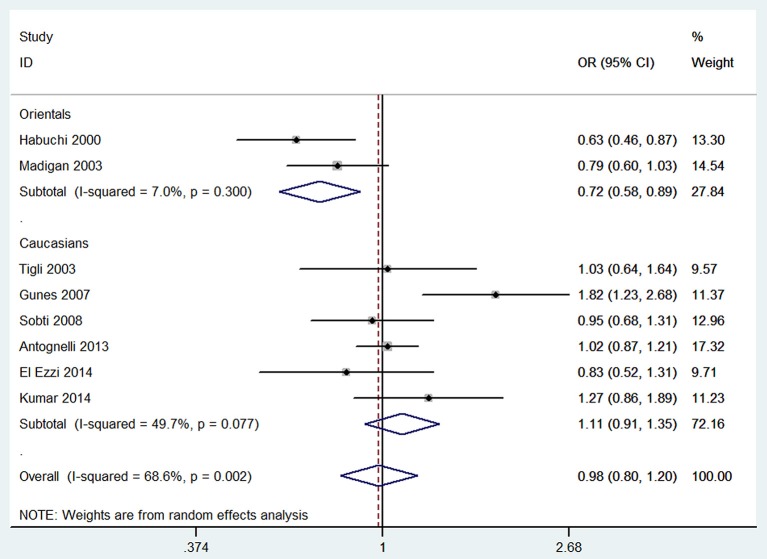
Forest plot of CYP17 rs743572 polymorphism and BPH risk in A1/A1 genotype.

Per-genotype approach showed that the OR_1_ (A1/A2 vs. A1/A1) and OR_2_ (A2/A2 vs. A1/A1) were 0.99 (95% CI: 0.79–1.25) and 0.97 (95% CI: 0.62–1.53), respectively. OR_1_ was homogeneous (*I*^2^ = 29.2%, *P* = 0.190), whereas OR_2_ was shown to be high heterogeneous across studies (*I*^2^ = 93.9%, *P* < 0.001). The results of sensitivity analyses were similar to those of the overall analysis. Subgroup analysis showed that A2/A2 genotype carriers had ~47% (OR = 0.53, 95% CI: 0.34–0.84) lower risk of developing BPH than individuals with A1/A1 genotype in Orientals and the heterogeneity was mild (*I*^2^ = 17.2%, *P* = 0.272) (Figure 2). No significant association was observed in Caucasians.

According to the model-free approach, the estimated λ value was 0.42 (95% CI: −0.01 to 0.85), suggesting that a co-dominant genetic model (A2/A2 vs. A1/A1 and A1/A2 vs. A1/A1) of inheritance was most likely. Table 4 shows all analyses of the estimation of genetic effect of CYP17 polymorphism on BPH.

### Publication Bias

**Figure 3:**
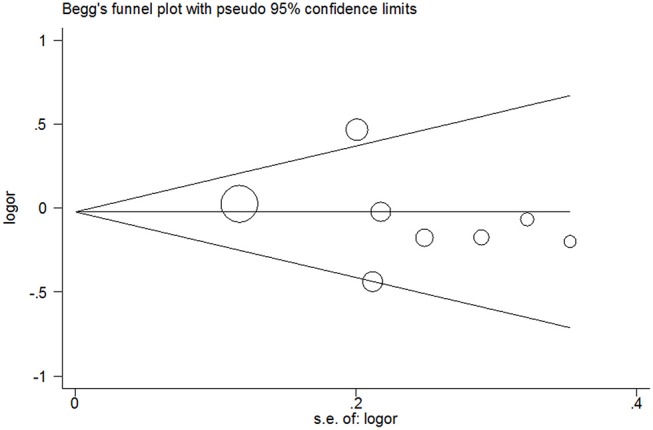
Begg's funnel plot for per-allele model.

**Figure 4:**
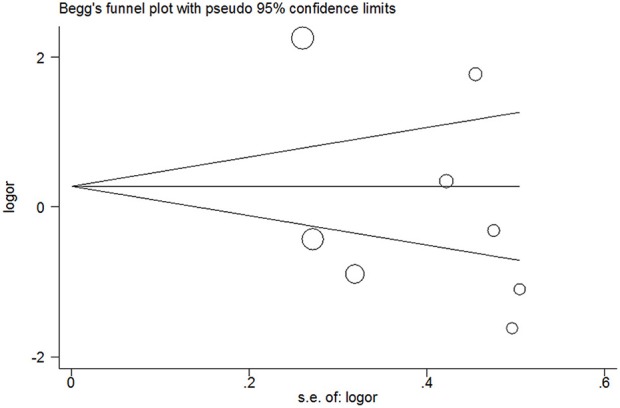
Begg's funnel plot for A1/A2 vs. A1/A1 model.

**Figure 5:**
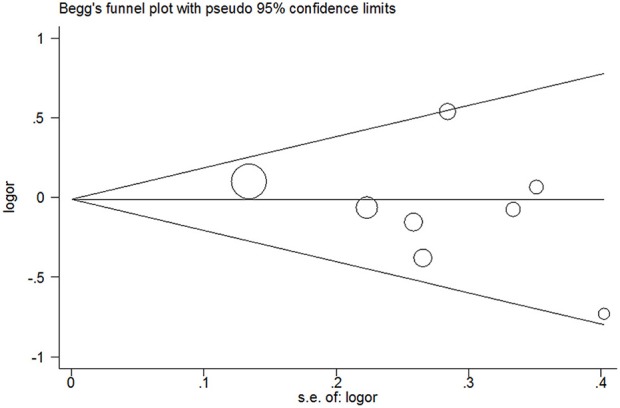
Begg's funnel plot for A2/A2 vs. A1/A1 model.

Publication bias was not detected since Begg's funnel plot for per-allele model (Figure 3), OR_1_ (Figure 4), and OR_2_ (Figure 5) all suggested symmetry of gene effects for all comparisons. The Egger's test also demonstrated the aforementioned results (*P* = 0.486 for A2 vs. A1; *P* = 0.345 for A2/A2 vs. A1/A1; *P* = 0.343 for A1/A2 vs. A1/A1).

## Discussion

BPH is a multifactorial and complex disease, and genetic effects have been considered as an important element in its etiology (Zeng et al., 2014). Many studies reported the effects of CYP17 rs743572 polymorphism on the susceptibility to BPH. In 2000, for the first time, Habuchi et al. (2000) suggested that CYP17 gene A1/A1 genotype increased risk of BPH with a gene dosage effect compared with A2/A2 genotype in Japanese population. However, subsequent studies did not achieve the same or similar results, and the true association between CYP17 gene polymorphism and BPH susceptibility was still controversial. In the present meta-analysis, we comprehensively summarized the evidence of the association profiles of CYP17 rs743572 polymorphism in BPH, and we observed no significant association between reported CYP17 rs743572 polymorphism and BPH in overall population and Caucasians. But we found a decreased risk of CYP17 polymorphism and BPH in Orientals. Therefore, the current available evidence suggests that CYP17 rs743572 polymorphism may decrease the risk of BPH in Orientals. A per-allele approach documented that A2 allele carriers had a ~28% lower risk of BPH than individuals with A1 allele in Orientals. A model-free approach suggested that the genetic model of inheritance was a co-dominant model, and the A2/A2 mutant homozygote was associated with a roughly 47% lower risk of developing BPH, compared with A1/A1 wild homozygote in Orientals. However, there was no such significant association in Caucasians. We assume that this difference might be attributed to the difference of MAF (56% in Orientals and 37% in Caucasians) between these two ethnicities.

There is a systematic review which evaluates the association between CYP17 rs743572 polymorphism and lower urinary tract symptoms in men with five case-control studies (Cartwright et al., 2014). Even though the results overall are the same, the additional three studies are worthwhile. In addition, our meta-analysis uses a more advanced method for estimating gene-disease associations, i.e., multivariate meta-analysis, which considers the within-study pairwise correlation of genotype contrast compared with the bivariate meta-analysis (Bagos, 2008). Moreover, a biological justification for the choice of the genetic model was performed. Our study also suggests certain implications for future research, and many details of this should be documented subsequently. First, certain identified risk factors (Matzkin and Soloway, 1993; Sea et al., 2009; Parsons, 2011) (such as age, androgen, and unhealthy lifestyles) of BPH need to be reported and adjusted in the original studies; therefore, we can justify the nature gene effects. Third, if the CYP17 rs743572 polymorphism is a factor which may decrease BPH susceptibility in Orientals, the causal relation and mechanism should be investigated. Furthermore, if the role of CYP17 rs743572 polymorphism is only a disease marker rather than a susceptibility or protective gene for BPH in Caucasians, the real risk factor should be identified.

Like every meta-analysis, our study also should be interpreted with caution due to certain inherent limitations of meta-analysis (Tikkinen et al., 2013; Yan et al., 2014; Zeng et al., 2014, 2015b,c; Gacci et al., 2015). First, the heterogeneity could distort the results of meta-analysis. In our study, the heterogeneity might be the most important limitation. There was moderate to high heterogeneity in the A2 vsA1 genetic model and A2/A2 vs. A1/A1 genetic model. Subgroup analysis showed that the heterogeneity might be derived from ethnicity, with heterogeneity detected in subgroup results for Caucasians but not for Orientals. Second, the biggest concern with all meta-analysis is the reliance on the available subjects of published papers, which may exaggerate the true effect due to publication bias. We cannot eliminate the possibility of publication bias even though we did not detect any publication bias using both Begg's funnel plot and Egger's test. Moreover, meta-analyses are prone to numerous potential biases, such as measurement error and genotyping error, not only the aforementioned publication bias. Third, our study collected data from only Caucasians and Orientals, and the results of the present systematic review and meta-analysis will only be applicable to these two ethnicities and so the external validity of our study has been restricted. Additionally, we could not perform haplotype analyses due to insufficient data of included studies, and the results may be spurious due to the haplotype gene effect (Niu et al., 2015). Ultimately, the relatively small sample size and the corresponding relatively low statistical power may limit effective detection of the true gene effect. Only two studies were reported for Oriental population and six for Caucasian population. Therefore, further studies with high quality and large sample size should be carried out.

In conclusion, our systematic review and meta-analysis provided a refined current evidence of CYP17 rs743572 polymorphism in BPH for the first time, and the results suggest that the CYP17 rs743572 polymorphism might be a protective gene for BPH in Orientals. Since the pooled sample size was small, the conclusion should be interpreted with caution and further studies with large sample size and high quality are warranted.

## Author Contributions

X-HW, X-TZ, and HW designed the study, participated in the acquisition of data, and helped draft the manuscript. HW and CF participated in the acquisition of data, summarized the collected evidence, and helped draft the manuscript. HW and P-LG performed the meta-analysis and helped draft the manuscript. X-HW, X-TZ, and Y-HJ revised the manuscript and helped coordinate the research. All authors read and approved the final manuscript.

### Conflict of Interest Statement

The authors declare that the research was conducted in the absence of any commercial or financial relationships that could be construed as a potential conflict of interest.
